# Supplementary material for: Causal Relationship between Aging and Anorexia Nervosa: A White-Matter-Microstructure-Mediated Mendelian Randomization Analysis
Source: Biomedicines. 2024 Aug 16;12(8):1874. doi: 10.3390/biomedicines12081874 (PMC11351342; doi:10.3390/biomedicines12081874)
Supplement: Supplementary file 1 [file biomedicines-12-01874-s001.zip › Supplementary Figure/Figure S4/Singe SNP plot of MR analysis for TL to ubm-b-2088.pdf]

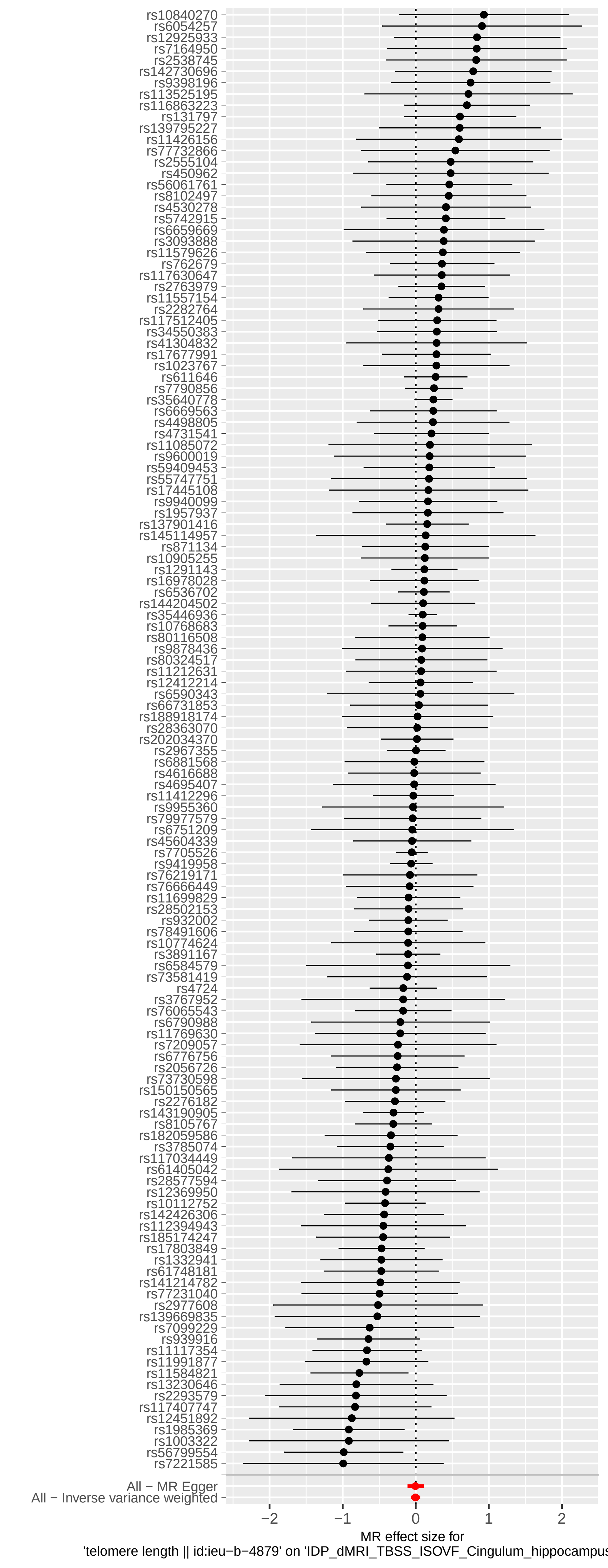
rs10840270
rs6054257
rs12925933
rs7164950
rs2538745
rs142730696
rs9398196
rs113525195
rs116863223
rs131797
rs139795227
rs11426156
rs77732866
rs2555104
rs450962
rs56061761
rs8102497
rs4530278
rs5742915
rs6659669
rs3093888
rs11579626
rs762679
rs117630647
rs2763979
rs11557154
rs2282764
rs117512405
rs34550383
rs41304832
rs17677991
rs1023767
rs611646
rs7790856
rs35640778
rs6669563
rs4498805
rs4731541
rs11085072
rs9600019
rs59409453
rs55747751
rs17445108
rs9940099
rs1957937
rs137901416
rs145114957
rs871134
rs10905255
rs1291143
rs16978028
rs6536702
rs144204502
rs35446936
rs10768683
rs80116508
rs9878436
rs80324517
rs11212631
rs12412214
rs6590343
rs66731853
rs188918174
rs28363070
rs202034370
rs2967355
rs6881568
rs4616688
rs4695407
rs11412296
rs9955360
rs79977579
rs6751209
rs45604339
rs7705526
rs9419958
rs76219171
rs76666449
rs11699829
rs28502153
rs932002
rs78491606
rs10774624
rs3891167
rs6584579
rs73581419
rs4724
rs3767952
rs76065543
rs6790988
rs11769630
rs7209057
rs6776756
rs2056726
rs73730598
rs150150565
rs2276182
rs143190905
rs8105767
rs182059586
rs3785074
rs117034449
rs61405042
rs28577594
rs12369950
rs10112752
rs142426306
rs112394943
rs185174247
rs17803849
rs1332941
rs61748181
rs141214782
rs77231040
rs2977608
rs139669835
rs7099229
rs939916
rs11117354
rs11991877
rs11584821
rs13230646
rs2293579
rs117407747
rs12451892
rs1985369
rs1003322
rs56799554
rs7221585
All − MR Egger
All − Inverse variance weighted
−2
−1
0
1
2
MR effect size for
'telomere length || id:ieu−b−4879' on 'IDP_dMRI_TBSS_ISOVF_Cingulum_hippocampus
